# Peer Review of “Assessing the Limitations of Large Language Models in Clinical Practice Guideline–Concordant Treatment Decision-Making on Real-World Data: Retrospective Study”

**DOI:** 10.2196/84175

**Published:** 2025-11-03

**Authors:** Reenu Singh

**Affiliations:** 1 Indian Institute of Management Mumbai, Vihar Lake Rd, Mumbai, India, 91 022 2803 5200

**Keywords:** large language model, foundation model, reasoning model, treatment decision-making, aortic stenosis, clinical practice guidelines, medical data processing

*This is the peer-review report for “Assessing the Limitations of Large Language Models in Clinical Practice Guideline–Concordant Treatment Decision-Making on Real-World Data: Retrospective Study.”*

## Round 1 Review

### Specific Comments

#### Major Comments

1. To improve the discussion on bias in large language models (LLMs) for clinical decision-making, the study [1] should include the following aspects:

If LLMs are trained predominantly on Western medical literature or specific demographic groups, their recommendations may not generalize well to diverse patient populations. If the data used to fine-tune the model lack representation from certain ethnic, gender, or socioeconomic groups, the artificial intelligence may produce recommendations that are not universally applicable. Even with a diverse dataset, biases can arise due to model architecture, reinforcement learning strategies, or human-in-the-loop feedback mechanisms that shape model responses.

2. What datasets were used? If real patient data were used, specify its source (eg, electronic health records, clinical trial data, or synthetic datasets). Provide the total number of cases or records used for testing the LLMs. If synthetic data were generated, describe the method used to create the data. Were diverse age groups, genders, and ethnic backgrounds represented? A lack of diversity in data can affect the generalizability of results.

3. What datasets were used? If real patient data were used, specify its source (eg, electronic health records, clinical trial data, or synthetic datasets). Provide the total number of cases or records used for testing the LLMs. If synthetic data were generated, describe the method used to create the data. Were diverse age groups, genders, and ethnic backgrounds represented? A lack of diversity in data can affect the generalizability of results.

The study’s impact can be significantly enhanced by addressing the following challenges: Raw medical reports often include free-text narratives, physician notes, abbreviations, and inconsistencies, requiring advanced natural language processing techniques such as entity recognition, text normalization, and standardization. These reports may also contain irrelevant information, redundancies, or nonessential clinical details. Effective preprocessing is essential to filter out unnecessary content while preserving critical medical insights. A key consideration is how to optimize this preprocessing to mitigate these challenges efficiently.

4. The study’s impact can be significantly enhanced by addressing the following challenges: Raw medical reports often include free-text narratives, physician notes, abbreviations, and inconsistencies, requiring advanced natural language processing techniques such as entity recognition, text normalization, and standardization. These reports may also contain irrelevant information, redundancies, or nonessential clinical details. Effective preprocessing is essential to filter out unnecessary content while preserving critical medical insights. A key consideration is how to optimize this preprocessing to mitigate these challenges efficiently.

## Round 2 Review

1. The authors have addressed the comments satisfactorily.

## Abbreviations

LLM: large language model
